# Patient motives for contacting out-of-hours care in Denmark: a cross-sectional study

**DOI:** 10.1186/s12873-020-00312-3

**Published:** 2020-03-17

**Authors:** Linda Huibers, Anders H. Carlsen, Grete Moth, Helle C. Christensen, Ingunn S. Riddervold, Morten B. Christensen

**Affiliations:** 1 Research Unit for General Practice, Bartholins Alle 2, 8000 Aarhus, Denmark; 2 Emergency Medical Services, Telegrafvej 5, 2750, Ballerup, Denmark; 3 Danish Clinical Quality Program (RKKP), Frederiksberg Hospital, Ndr. Fasanvej 57, 2000, Frederiksberg, Denmark; 4 Prehospital Emergency Medical Services, Central Denmark Region, Olof Palmes Allé 34, 8200 Aarhus, N Denmark

**Keywords:** After-hours care, Primary health care, Emergency medical services, Help-seeking behaviour, Motivation, Patient safety, Emergency medicine

## Abstract

**Background:**

Patients in need of acute health care do not always contact the most suitable health care service provider. Contacting out-of-hours primary care for an urgent problem may delay care, whereas contacting emergency medical services for a non-urgent problem could ultimately affect patient safety. More insight into patient motives for contacting a specific health care provider may help optimise patient flows. This study aims to explore patient motives for contacting out-of-hours primary care and the emergency medical services in Denmark.

**Methods:**

We conducted a cross-sectional observational study by sending a questionnaire to patients contacting out-of-hours primary care and emergency medical services, both of which can be directly contacted by patients, in two of five Danish regions in 2015. As we aimed to focus on the first access point, the emergency department was not included. The questionnaire included items on patient characteristics, health problem and 26 pre-defined motives. Descriptive analyses of patient characteristics and motives were conducted, stratified by the two health care service providers. Factors associated with contacting each of the two service providers were explored in a modified Poisson regression analysis, and adjusted risk ratios were calculated.

**Results:**

Three key motives for contacting the two service providers were identified: ‘unpleasant symptoms’, ‘perceived need for prompt action’ and ‘perceived most suitable health care provider’. Other important motives were ‘need arose outside office hours’ and ‘wanted to talk to a physician’ (out-of-hours primary care) and ‘expected need for ambulance’ and ‘worried’ (emergency medical services). Higher probability of contacting the emergency medical services versus out-of-hours primary care was seen for most motives relating to own assessment and expectations, previous experience and knowledge, and own needs and wishes. Lower probability was seen for most motives relating to perceived barriers and benefits.

**Conclusions:**

Patient motives for contacting the two health care service providers were partly overlapping. The study contributes with new knowledge on the complex decision-making process of patients in need of acute health care. This knowledge could help optimise existing health care services, such as patient safety and the service level, without increasing health care costs.

## Background

Recent years have seen an increase in the number of contacts to the acute health care services. Crowding of patients at emergency departments (EDs), excessive demands on the emergency medical services (EMS) [1–3] and more frequent use of out-of-hours primary care are widespread in many countries [4]. The high demand may have several negative effects: high use of resources, increased health care costs and high workload for health care professionals. This development may further cause higher risk of errors, longer waiting times for patients and potential treatment delay, and lower job satisfaction [1, 5–11].

When experiencing a health problem, a patient is likely to request prompt medical assessment and may thus decide to contact a specific health care service. Apart from the specific health problem, other motives can influence the decision to contact a health care service outside office hours. Worry and a perceived urgent need to see a general practitioner (GP) are frequently mentioned as motives for contacting out-of-hours primary care-, but perceived lack of availability and accessibility of own GP also seems to play a role [12]. Prevention or ruling out of serious disease is another important motivation, specifically in parents of children [13]. Worrying and anxiety have also been identified as important motives for contacting EMS and EDs [3, 14]. The prospects of receiving fast help and getting easy access to diagnostic tests are known motives for contacting the ED, as are symptoms perceived as being too severe for assessment in primary care [14–17].

Two options for getting health care outside own GP’s office hours are offered in several countries: calling out-of-hours primary care or dialling the national emergency number (1–1-2 in Europe, 9–1-1 in the USA). The choice between these two should be based on the urgency level of the experienced health problem, but many other factors influence the patient’s decision. Consequently, patients do not always contact the most relevant health care service [12, 18–23] and thus do not get the most suitable care. Contacting primary care for an urgent health problem may delay the care and worsen the condition, whereas contacting the EMS for a non-urgent problem may ultimately affect patient safety outcomes due to work overload and overtreatment; this may also result in unnecessary use of resources.

More insight into patients’ motives for choosing specific out-of-hours health care services is important as new knowledge in this field could be used to make suitable adjustments of the existing health care services and to develop initiatives guiding the patients in choosing the most relevant service for their health problem. This may ultimately help reduce the workload in the out-of-hours health care services and increase the service level through better management of patient safety and reduced delay in the care for severely ill patients.

## Methods

### Aim

The aim of this study was to explore patient motives for seeking acute health care at out-of-hours primary care and the EMS and to investigate motives associated with contacting each of these two health care service providers.

### Design and setting

We conducted a cross-sectional observational study to explore patients’ motives for contacting out-of-hours care by sending a questionnaire to patients who had contacted out-of-hours –primary care and the EMS. Data was collected in two Danish regions, the Capital Region of Denmark in Copenhagen and the Central Denmark Region, during a two-week period in February–March 2015.

All citizens with fixed abode in Denmark are listed with a GP and have access to the public (tax-funded) health care system free of charge. GPs serve as gatekeepers to secondary care and are usually available on weekdays from 8 am to 4 pm. Denmark is divided into five regions; each of these regions is responsible for organising health care in their own region. Health care is provided by primary care (both daytime and -out-of-hours primary care), the EMS and secondary care (e.g. hospitals, EDs). Referral from either primary care or the EMS is required before an ED visit or hospital admission. Therefore, we did not include the ED. Direct use (i.e. self-referring) to out-of-hours primary care and the ED is low in Denmark, due to required telephone access.

In the Central Denmark Region, out-of-hours primary care is organised by GPs in large-scale cooperatives (GPCs). GPs perform telephone triage and deal with the presented problem by giving telephone advice or by referring the patient to a subsequent face-to-face consultation [24]. In Copenhagen out-of-hours primary care is an integrated part of the EMS, with medical helpline 1813 (MH-1813) serving as a dedicated entrance for non-urgent cases. Nurses perform the triage; they are supported by a computerised decision-support tool and the opportunity to consult a doctor (or hand over the call), but these doctors may also answer direct calls. Patients receive telephone advice or are referred to a face-to-face consultation.

In both regions, the EMS consists of an emergency medical coordination centre (EMCC) handling all 1–1-2 emergency calls. The EMCC is staffed by different types of health care professionals (nurses, paramedics and doctors for supervision), who asses the urgency level and decide on the suitable response, as indicated by the criteria-based dispatch protocol named the Danish Index for Emergency Care [25]. This protocol states 37 main dispatch criteria (symptoms) and divides calls into 5 levels of emergency.

### Study population

We included patients who contacted out-of-hours primary care or the EMS outside office hours (i.e. weekdays from 4 pm to 8 am, entire weekends and bank holidays), either themselves or by another person. The first contact with a health care professional within the study period was included for each patient. If a patient had a follow-up contact with the other out-of-hours health care service provider within the study period, we included only the first contact. Exclusion criteria were: contact during daytime, death at the time of dispatching questionnaires, address protection, living in an institution, tourists and other citizens with an invalid personal identification number (PIN) [26] and participation in one of the pilot studies. Patients aged 13–18 years were also excluded for confidentiality reasons. Moreover, for EMS contacts, we excluded patient transport ‘planned in advance’ and requests for an acute ambulance by health care professionals. Contacts with a bystander calling were also included, which is assessed more common for EMS contacts. A recorded message on the telephone waiting line informed patients calling the GPC and MH-1813 about the ongoing research project, and callers were given the opportunity to decline participation by pressing ‘9’.

### Development of questionnaire

First, a literature search was conducted, and existing questionnaires on patient motives were studied, resulting in an overview of factors related to decision-making in patients and prevailing motives for contacting out-of-hours care. Next, these factors and motives were categorised into a model for decision-making when contacting out-of-hours health care services. The model was based on Andersen’s Behavioural Model [27] and adapted to the Danish health care system. Several internal research meetings and an external expert feedback round were held, resulting in the final questionnaire (Additional file 1). Motives were measured by 26 predefined statements relating to the decision to contact out-of-hours care (Additional file 2). Respondents were asked to rate, on a 5-point Likert scale, the importance of each statement for the decision. Motives were grouped into: ‘own assessment and expectations’, ‘barriers and benefits’, ‘previous experience and knowledge’, and ‘needs and wishes’. We tested the questionnaire twice in the GPC waiting room and interviewed four patients in a general practice waiting room to ensure clarity and validity. Moreover, three small-scale pilot studies were conducted to enhance clarity, increase the response rate and enable a power calculation.

The final questionnaire included questions on patient characteristics, the health-related problem and the patient’s motives for contacting out-of-hours care. Patient characteristics included: age and sex, decision maker (patient himself/herself, family member, other known person or unknown person), ethnicity and marital status. Questions about the health-related problem included: main problem and duration. In addition, we included information extracted from the patient registration systems of the out-of-hours health care service providers: date and time of contact, patient’s PIN, type of contact and urgency level (only for EMS contacts). The PIN was used to calculate age and sex, search for duplicates and check the patient’s status (possible death) before sending the questionnaires.

### Data collection

A power calculation showed that we needed a study population of 400 respondents per health care service provider, each consisting of two units, to be able to detect a 10% difference in the importance of motives between the out-of-hours primary care and EMS, as well as between the GPC and MH-1813. Having obtained a response rate of 40% in our final pilot study, we aimed to send out 1000 questionnaires per health care unit. As we also aimed to compare motives for contacts regarding children and adults, we selected 1000 patients < 13 years and 1000 patients > 18 years for both the GPC and MH-1813. We selected 1100 patients rather than 1000 per EMS unit, as we expected a lower response rate and more exclusions due to high numbers of bystander calls and incorrect PINs. The data collection lasted 1 week for the two out-of-hours primary care service providers (GPC and MH-1813) and 2 weeks for the EMS due to differences in number of weekly patient contacts.

Data on calls were received twice a week, and questionnaires were dispatched within 4 days after the relevant out-of-hours contact to ensure vivid recall of contact details and the decision-making process. We randomly selected contacts from each health care unit, and the patient’s address and status (alive/deceased) was verified in the Civil Registration System. Invitation letter and paper questionnaire were sent to patients aged > 18 years and registered guardians for patients aged < 13 years, including a link and login credentials to a web-based version of the questionnaire. One reminder was sent after 2 weeks. Questionnaires were sent to the patients, including for contacts initiated by bystanders. These bystander calls were assessed more common for EMS contacts, with a.

### Statistical analyses

Descriptive analyses were performed to identify the main characteristics of contacts and respondents, stratified by health care service. Motives were dichotomised into ‘not important’ (‘not relevant’, ‘no importance’, ‘little importance’, ‘some importance’) and ‘important’ (‘important’ and ‘very important’), and the percentage of importance per motive was estimated for each of the two health care services. Aiming to identify motives for contacting the EMS (as opposed to contacting out-of-hours primary care) and to obtain risk ratios (RR), we applied the modified Poisson regression model for all contacts and stratified for children and adults [28]. The resulting RRs were presented along with 95% confidence intervals. Moreover, a non-response analysis was conducted. Stata statistical software, version 14, was used (StataCorp LP, College Station, TX, USA).

## Results

### Study population

**Fig. 1:**
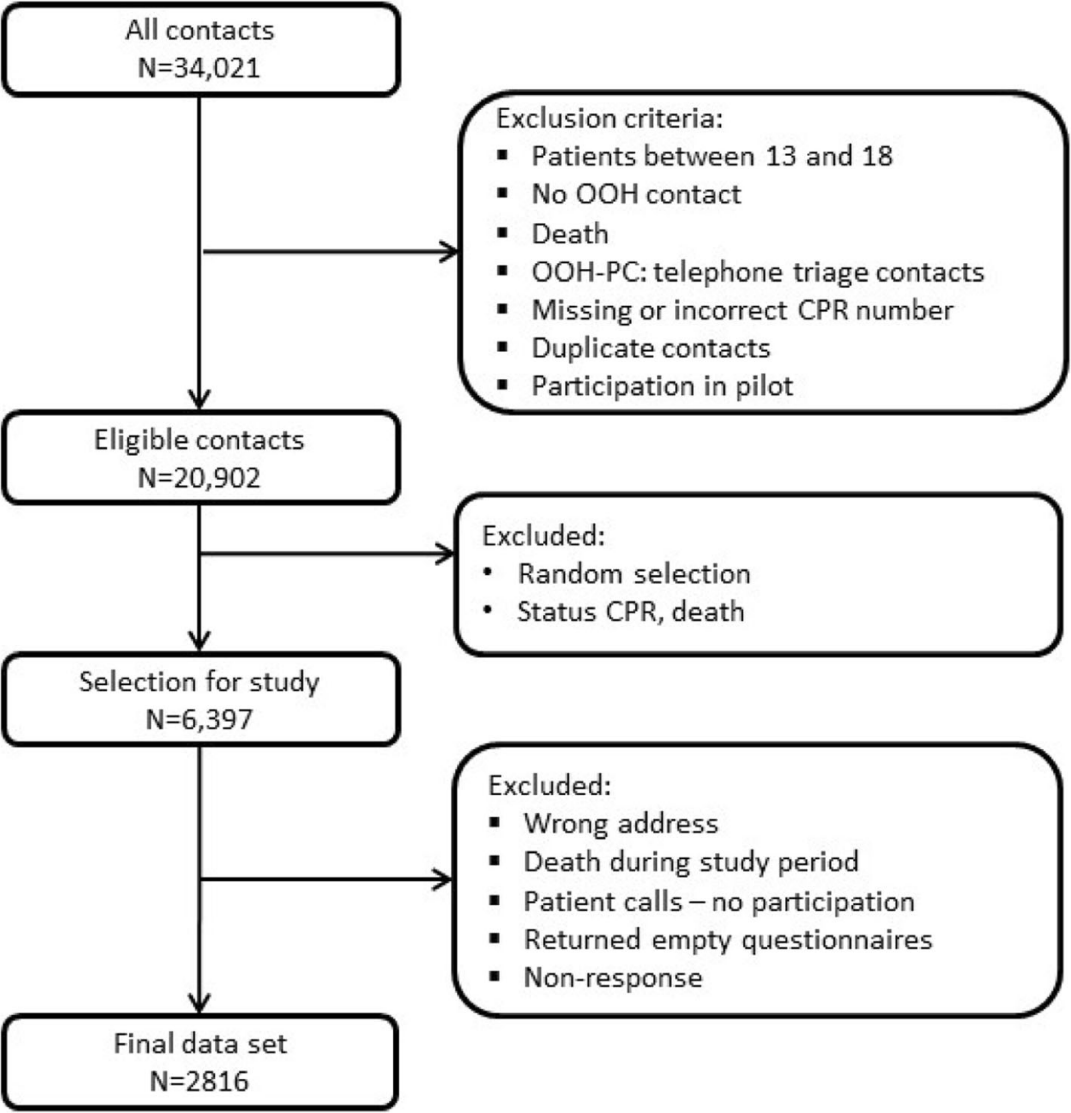
Flowchart of study population

Flowchart 1 shows the selection of the population. The final response rate was 44.9% (out-of-hours primary care: 48.1% for children and 46.2% for adults; EMS: 52.1% for children and 40.4% for adults) (Fig. 1).

### Characteristics of respondents

Respondent characteristics are shown in Table 1. The percentage of contacts ending with telephone advice was higher in out-of-hours primary care (children: 56.3%; adults: 52.8%) than in the EMS (children: 14.5%; adults: 10.8%). Furthermore, differences were found for average age of adult patients (out-of-hours primary care: 49.0 years; EMS: 61.6 years) and symptom duration (out-of-hours primary care: 1–24 h; EMS: < 1 h). Furthermore, adults contacting out-of-hours primary care had more often high educational level, were more often employed and reported more often to have good health status than adults contacting the EMS.

**Table 1:** Description of population (%)

	OUT-OF-HOURS-PRIMARY CARE		EMS	
	Children (*N* = 961)	Adults (*N* = 910)	Children (*N* = 89)	Adults (*N* = 886)
Characteristics of contact				
Time of contact, weekend^1^	56.8	57.5	58.4	55.0
Type of contact, telephone advice	56.3	52.8	14.5	10.7
Age patient (mean)	4.0	49.0	3.9	61.6
Main health problem, self-assessed	*n* = 1342	*n* = 1084	*n* = 134	*n* = 996
- Symptoms/complaints	77.9	68.1	76.9	69.5
- Infections	7.2	8.8	0.7	0.8
- Trauma	4.9	7.6	13.4	17.8
- Other	10.1	15.6	9.0	11.9
Duration of symptom				
- < 1 h	14.4	16.1	94.3	66.1
- 1–24 h	52.1	52.0	4.6	30.1
- > 24 h	33.4	31.9	1.2	3.8
Decision maker				
- Patient/parent	97.3	84.5	89.9	45.0
- Family/other	2.7	15.6	7.9	46.9
- Unknown	0.0	0.0	2.3	8.1
Characteristics of patient/guardian^2^				
Age (years)				
- 18–39	73.9	36.0	74.4	16.8
- 40–64	26.1	40.6	24.4	31.1
- > 64	0.0	23.4	1.2	52.1
Sex, female	80.5	62.5	72.1	47.2
Education				
- Low	4.9	15.0	10.8	26.7
- Middle	34.2	47.7	31.3	45.6
- High	60.9	37.3	57.8	27.7
Ethnicity				
- Native	85.4	88.9	78.3	85.7
- Western migrants	7.6	6.0	10.8	8.9
- Non-western migrants	7.0	5.1	10.8	5.5
Marital status, single	9.3	30.6	21.4	39.3
Employment, not working	26.9	45.5	28.9	71.2
Health status, self-assessed, poor	5.3	23.4	6.0	36.6

^1^Day: weekend is from Friday 4 p.m. until Monday 8 a.m., week is Monday to Thursday from 4 p.m. to 8 a.m. the next day; ^2^For children, background information concerns parent/caregiver; Number of missing values varies per variable

A non-response analysis (not in Table) showed a statistically significantly different mean age between respondents and non-respondents who had contacted out-of-hours primary care (children: 3.99 vs. 3.59 years, adults: 49.0 vs. 41.0 years) and the EMS (adults: 61.6 vs. 53.5 year). Out-of-hours primary care non-respondents were significantly more often female than respondents (51.6% vs. 48.4%). Calls from adult respondents to the EMS significantly more often resulted in an ambulance dispatch than calls from non-respondents (42.2% vs. 31.5%). No difference was found for weekday, time of contact or contact type in out-of-hours primary care (face-to-face vs. telephone). Only few callers refrained from typing in their PIN when calling out-of-hours primary care E (children: 0.6%; adults 4.4%). At MH-1813 and GPC, approximately one in four of callers chose to decline participation by pressing 9 (MH-1813: children: 24.2%, adults: 26.0%; GPC: children: 23.2%, adults 29.5%).

### Motives for contacting

The three most important motives were ‘unpleasant symptoms’, ‘perceived need for prompt action’ and ‘perceived most suitable health care provider’ (Table 2). The fourth and fifth most important motives were ‘need arose outside office hours’ and ‘wanted to talk to a physician’ for out-of-hours primary care and ‘expected need for ambulance’ and ‘worried’ for the EMS. For both health care service providers, the motives ‘own GP not accessible during daytime’ and ‘own GP no time available soon enough’ were also regularly mentioned (about 10%).

**Table 2:**
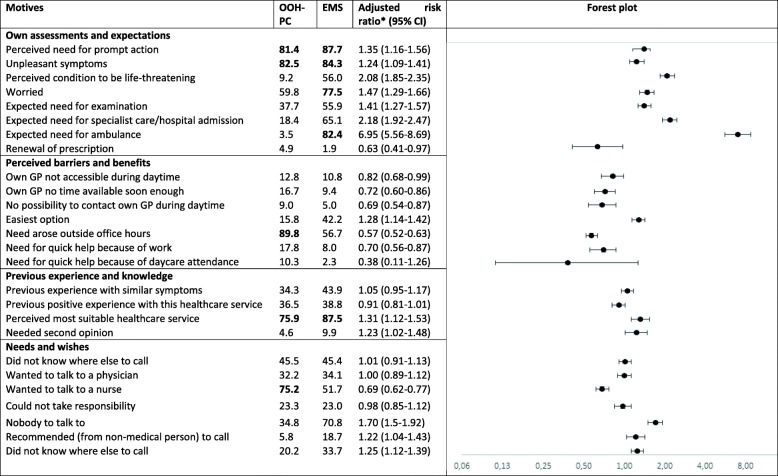
Motives for contacting out-of-hours health care, stratified per setting, with the five most important motives marked in bold (% according to importance) and adjusted risk ratio for motives associated with out-of-hours primary care contact versus EMS (RR, 95% CI)

Adjusted for duration of symptom, weekend/weekday, decision-maker characteristics (age, sex, highest education level, job status, ethnicity, self-assessed health)

### Factors associated with contacting out-of-hours primary care versus EMS

Table 2 presents the adjusted RRs for motives with higher probability of contacting the EMS versus contacting out-of-hours primary care. Most motives were related to own assessment and expectations, e.g. ‘perceived condition to be life-threatening’ (RR = 2.08, 95% CI: 1.85–2.25) and ‘expected need for ambulance’ (RR = 6.95, 95% CI: 5.56–8.69). Some motives were related to previous experience and knowledge, e.g. ‘perceived most suitable health care provider’ (RR = 1.31, 95% CI: 1.12–1-53) and ‘needed second opinion’ (RR = 1.23, 95% CI: 1.02–1.48). Other motives were related to own needs and wishes, e.g. ‘could not take responsibility’ (RR = 1.70, 95% CI: 1.50–1.92), ‘nobody to talk to’ (RR = 1.22, 95% CI: 1.04–1.43) and ‘recommended (from non-medical person) to call’ (RR = 1.25, 95% CI: 1.12–1.39). Some motives involved lower probability of contacting EMS versus out-of-hours primary care. Most of these motives were related to perceived barriers and benefits, e.g. ‘own GP no time available soon enough’ (RR = 0.72, 95% CI: 0.60–0.86), ‘no possibility to contact own GP during daytime’ (RR = 0.69, 95% CI: 0.54–0.87) and ‘wanted to talk to a physician’ (RR = 0.69, 95% CI: 0.62–0.77). The motive ‘renewal of prescription’ also had a lower probability (RR = 0.63, 95% CI: 0.41–0.97).

**Fig. 2:**
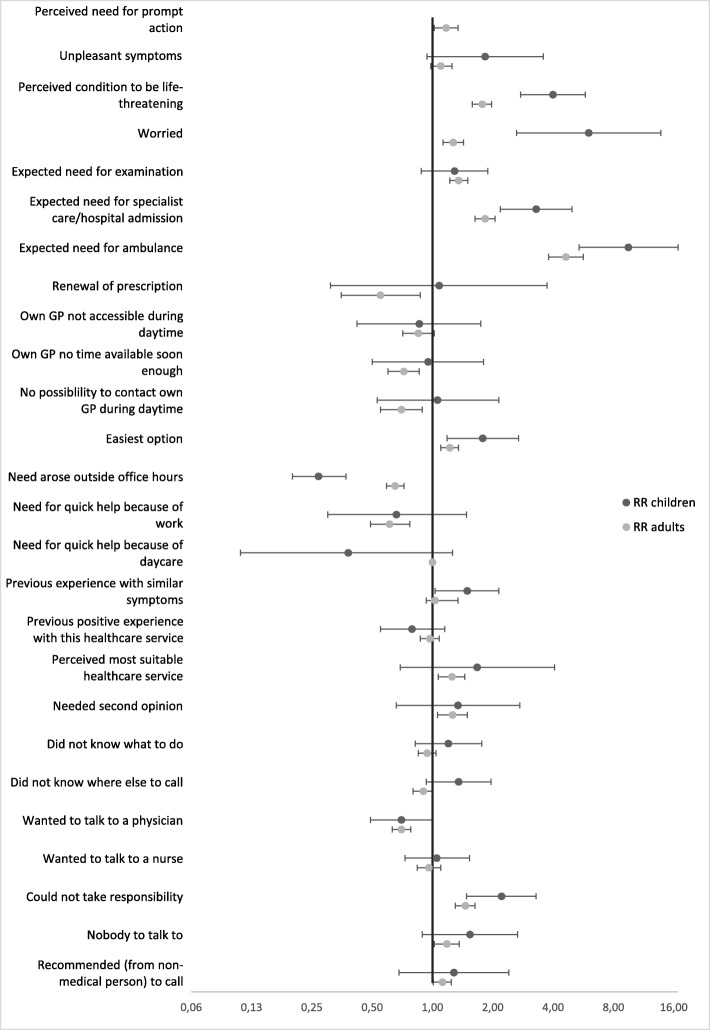
Forest plot: adjusted risk ratio for motives associated with out-of-hours primary care versus EMS contacts (children and adults). Adjusted for patient (adult or child), duration of symptom, weekend/weekday, decision-maker characteristics (age, sex, highest education level, job status, ethnicity, self-assessed health)

Figure 2 presents the adjusted RRs of motives associated with contacting either the EMS or out-of-hours primary care for health problems in children and adults. Significant differences were seen for ‘perceived condition to be life-threatening’ (children: RR = 3.99, 95% CI: 2.75–5.79; adults: RR = 1.77, 95% CI: 1.58–1.97), ‘worried’ (children: RR = 6.02, 95% CI: 2.63–13.80; adults: RR = 1.27, 95% CI: 1.13–1.43), ‘expected need for specialist care or hospital admission’ (children: RR = 3.29, 95% CI: 2.16–4.37; adults: RR = 1.83, 95% CI: 1.63–2.05) and ‘need arose outside office hours’ (children: RR = 0.27, 95% CI: 0.20–0.37; adults: RR = 0.65, 95% CI: 0.59–0.72).

## Discussion

### Key results

Compared with the EMS, adult callers to out-of-hours primary care were younger, more often female, were more often employed, more often had a high educational level and self-reported more often good health status. Several motives were associated with *higher* probability of contacting the EMS versus contacting out-of-hours primary care. Most of these motives related to own assessment and expectations, but some motives related to previous experience and knowledge or needs and wishes. Motives associated with *lower* probability were mostly related to perceived barriers and benefits. Only four motives associated with contacting the EMS versus out-of-hours primary care differed significantly between children and adults.

### Comparison with existing literature

To our knowledge, no previous studies have compared patients calling out-of-hours primary care and the EMS within one design, but several studies have investigated patients calling out-of-hours primary care [29–32]. In line with these single-service studies and clinical experiences, we found some differences between patients calling out-of-hours primary care and patients calling the EMS. As in other studies, women more often than men contacted out-of-hours primary care, and a considerable part of calls to out-of-hours primary care concerned children [29–32]. Two Danish studies on EMS contacts reported similar percentages of calls made by women, but these studies found a slightly lower mean age than found in our study [33, 34]. This difference is likely to be due to our stratification into groups (children and adults) and exclusion of patients aged 13–18 years.

The most important motives found in our study for contacting out-of-hours primary care were partly in line with other studies. Worry and need for reassurance are frequently mentioned motives in out-of-hours primary care, as also found in other studies [12, 35, 36]. Kallestrup et al. reported that symptom relief was an important motive in about one third of contacts to out-of-hours primary care [35]. Parents of Dutch children with fever have been found to contact to get reassurance from a professional [37], and a considerable part of Dutch patients have reported a perceived need to see a GP [12]. We found that perceived availability and accessibility of own GP play a role for a minority of patients, as found by Keizer et al. [12].

As far as we know, only few previous studies have focused on motives for contacting the EMS [3, 38]. The existing studies found motives similar to the motives identified in our study. Booker et al. also reported that worry and anxiety were two important motives [3]. Furthermore, they found that callers with care responsibilities tend to contact the health care service that is expected to provide the promptest response, as decision making is driven by lower tolerance of perceived risk [3]. This result is closely related to our finding that callers could not take responsibility. In line with our findings, Ahl et al. found that the need for immediate help was an important criterion for deciding to contact ambulance care [38], and patients are aware that ambulance services provide a quick response [3]. In addition, some of the identified motives seem to match those found for patients self-referring to the ED, such as easier access to diagnostic tests and symptoms perceived to be too severe to be handled by the GP [14–17].

Patients frequently contacting out-of-hours primary care and the EMS [1–4] do not always choose the most suitable health care service provider [23, 31, 39–43], which could cause delay of care, overcrowding, overtreatment and overuse of resources. It is important to acknowledge the patient’s role in the complex decision-making process when facing an acute health care problem. The traditional focus on medical relevance should instead be directed towards ways of assisting the patient in the decision-making process (patient-centredness). Contacting a less suitable health care service may occur because of little knowledge of available acute health care services and/or of suitable care for specific symptoms. The identified motives for calling the EMS (i.e. expectation of prompt diagnostics or need for specialist care, hospital admission or ambulance dispatch) reflect the patients’ own assessment of symptoms and own care expectations, which seem in line with the more acute character of EMS. Patients make this conclusion on the basis of their knowledge of the health care system [36] and of the disease presentation.

The availability and accessibility of own GP and personal barriers (‘no opportunity to call the GP’, ‘need for quick help because of work/daycare’) could be areas of improvement, as these motives were mentioned by patients at both health care services. Several other studies have shown an association between the accessibility of own GP and use of out-of-hours primary care [36, 44]; this association may also be relevant for EMS contacts. In our study, most patients stated that their need emerged outside the opening hours of their own GP. The need to contact health care may truly have appeared outside the opening hours [45], but the answers could also have been biased by social desirability. Parents may find it difficult to decide whether to contact out-of-hours primary care for a health problem occurring outside office hours or wait until the opening hours of own GP [36], whereas GPs may find that patients have a low threshold for contacting out-of-hours care [46].

### Strengths and limitations

We conducted a large-scale study exploring patient characteristics and motives to contact the out-of-hours health care services in two Danish regions, with parallel data collection at out-of-hours primary care and the EMS. The developed questionnaire was found to have good face and content validity, and three small-scale pilot studies ensured further optimisation. Based on literature and feedback from experts and patients, we acquired a thorough overview of relevant patient motives for contacting out-of-hours care. Yet, our studies also had some limitations.

We cannot rule out selection bias, even though our response rate (44.9%) was acceptable for this type of study. The non-response analysis showed that some characteristics differed between our respondents and non-respondents. This may have influenced our results on important motives for contacting out-of-hours care, as some motives related to specific patient groups. A considerable drop-out rate was seen at out-of-hours primary care as some patients declined participation. We predefined 26 motives that were considered relevant for contacting out-of-hours care and asked the respondents to assess their importance. Thus, the patients could point out multiple relevant motives, without ranking the most important ones. This approach allowed us to consider the impact of all motives, including the ones perceived as less important, which is relevant for the understanding of the decision-making process in patients. Our list of 26 predefined motives may not be complete, thus introducing some bias. Yet, as our list was defined after an extensive procedure, we expect this bias to be minimal. Primarily, we studied the motives underlying decision-making, without assessing suitableness or patient outcome. However, we could not rule out social desirability bias, as patients may have wanted to give suitable and acceptable motives for their contact and the received health care. We included all contacts, including bystander calls to EMS. As questionnaires were answered by patients, some information bias may appear for bystander calls. Yet, most calls were made by family members or other known bystanders. Finally, generalisation of findings to other populations in similar health care systems should be made with caution, as the access to both out-of-hours primary care and the EMS is free of charge in Denmark and access is by telephone call.

### Recommendations for future research and clinical practice

Our study contributes to understanding the complex decision-making process of patients in need of acute health care. This knowledge may contribute to suitable adjustment of the existing health care services, aiming to optimise patient safety and service level without increasing health care costs. Previous studies have found that patients do not always access the most suitable service, which could be caused by a range of factors. Future studies focusing on the identified motives seem relevant, such as the importance of the availability and accessibility of own GP for the decision to contact out-of-hours primary care and the potential effect of ensuring better availability and accessibility. Furthermore, an international comparison could be interesting, giving the opportunity to study different organizational and health care system factors in relation to motives for help seeking. Moreover, patients could be assisted by public information campaigns on available health care services and target groups, and the effects of creating one access point to acute care could be explored.

## Conclusions

We identified five key patient motives for seeking acute health care at out-of-hours primary care and the EMS; some of these motives were partly overlapping (i.e. ‘unpleasant symptoms’, ‘perceived need for prompt action’ and ‘perceived most suitable health care provider’). Several factors were associated with contacting OOH-PC versus EMS. Most motives relating to own assessment and expectations, previous experience and knowledge, and own needs and wishes were related to a higher probability of contacting EMS versus out-of-hours primary care, whereas most motives relating to perceived barriers and benefits were related to a lower probability. This knowledge could contribute to adjustments of the current health care services, with the aim to optimise patient safety and service level without increasing health care costs.

## Supplementary information

**Additional file 1.** Questionnaire “Why did you contact the out-of-hours service?”

**Additional file 2.** Abbreviation list of the motives.

## Data Availability

The datasets used and analysed as part of this study are available from the corresponding author on reasonable request.

## Abbreviations

1–1-2: The emergency number 1–1-2
95% CI: 95% confidence interval
ED: Emergency department
GP: General practitioner
GPC: GP cooperative
MH-1813: Medical helpline 1813
RR: Risk ratio
