# Supplementary material for: Patient motives for contacting out-of-hours care in Denmark: a cross-sectional study
Source: BMC Emerg Med. 2020 Mar 17;20:20. doi: 10.1186/s12873-020-00312-3 (PMC7079359; doi:10.1186/s12873-020-00312-3)
Supplement: Supplementary file 1 — Additional file 1. Questionnaire “Why did you contact the out-of-hours service?” [file 12873_2020_312_MOESM1_ESM.docx]

**Appendix 1.** Questionnaire “Why did you contact the out-of-hours service?”

The questionnaire was sent to callers contacting out-of-hours primary care and the emergency medical service. Slightly different versions were used; the name of the specific service (one of the two) was stated throughout the questionnaire, and the wording of response categories in the version for adults differed slightly from the version for children. For practical reasons, a general version of the questionnaire was translated. In this general version, the *out-of-hours service* covered both out-of-hours primary care and the emergency medical service, and all response categories were included. Item 8 was presented in two different versions targeting the two difference service providers, as this item concerned extensive differences.

**INTRODUCTION**

What is your age?

Age: ____ years

What is your sex?

Male

Female

1. Did you (yourself) make the decision to call the out-of-hours service? *(Please tick only one box)*

- Yes, I made the decision or participated in making the decision
- No, someone else/others made the decision:
  - Family member: ________________ *(Please state whom, e.g. wife, son, daughter)*
  - Someone else: _____________________ *(Please state whom, e.g. friend, neighbour, colleague)*
  - Unknown person

If you did not participate in making the decision to contact the out-of-hours service, we would still like you to answer the questionnaire although there may be questions that you will not be able to answer. In any case, we ask you to return the questionnaire.

**THE PROBLEM THAT MADE YOU SEEK HELP**

What was the most important (health) problem that made you call the out-of-hours service?

________________________________

How long had the problem in question lasted before you called the out-of-hours service? *(Please only tick one box)*

Less than 1 hour

1-5 hours

6-24 hours

More than 24 hours

Not relevant/don’t know

What did you do yourself to solve the problem before contacting the out-of-hours service? *(You may tick more than one box)*

Nothing, as I soon decided to contact the out-of-hour service

Wait and see for a period of time

Asked family/friends for advice

Searched for help/information on the Internet

Self-care/first aid (e.g. clean wounds, take painkiller, use usual asthma medication)

Tried in vain to contact my own GP earlier

Was in contact with my own GP

Tried in vain to contact out-of-hours primary care

Was in contact with 112/out-of-hours primary care

Other. *Please describe:* ________________________________

Not relevant/don’t know

What did you expect to get out of calling the out-of-hours service? *(You may tick more than one answer)*

- Advice on what I could do myself
- Information about what was wrong with me
- Assessment of how serious the problem was
- Decision on whether treatment was required
- Change/renewal of prescribed medication that I was already being treated with
- Examination (e.g. blood test, throat swab, X-ray)
- Treatment (e.g. new medication, wound treatment)
- Referral to emergency department (e.g. for an injury)
- Have an ambulance sent
- Admission to hospital
- Other. *Please describe:* ________________________________

Not relevant/don’t know

What came out of your contact to the out-of-hours service? *(Please tick only one box)* (version for out-of-hours primary care service provider)

Telephone consultation (contact ended by telephone with advice/treatment)

Telephone consultation (contact ended by telephone with direct referral to the emergency department)

Clinic consultation at out-of-hours primary care

Home visit of a GP on duty

Other. *Please describe*: ________________________________

What came out of your contact to the out-of-hours service? *(Please tick only one box)*

(version for emergency medical service)

Telephone consultation (NO ambulance sent, contact ended by telephone advice/treatment, e.g. referral to out-of-hours primary care or emergency department)

Ambulance sent – transport to hospital

Ambulance sent – NO transport to hospital

Other. *Please describe*: ________________________________

1. Which type of treatment/advice did you get? *(You may tick more than one box)*

- Advice and information
- Recommended use of over-the-counter medication (e.g. nose drops, painkillers)
- Recommendation to contact own GP in the daytime
- Recommendation to contact the out-of-hours services again if my condition did not improve, or I got worse
- Examination (e.g. blood test, throat swap, X-ray)
- Change/renewal of prescribed medication that I was already being treated with
- Treatment (e.g. new medication, wound treatment)
- Referral to emergency department
- Ambulance was sent
- Admission to hospital
- Other. *Please describe:* ________________________________
- Not relevant/don’t know

**YOUR MOTIVATION FOR CONTACTING THE OUT-OF-HOURS SERVICE**

There can be several reasons for choosing to contact the out-of-hours service. A health problem can occur outside the opening hours of your own general practitioner (GP). In other cases, a problem may have lasted for several hours or days, but there can still be good reasons to call the after-hours doctor instead of your own GP. Because of these differences, some of the questions below may not always make sense. In those cases, you may tick the response "Not relevant".

The next questions are of particular importance for the study. Therefore, it is important that you answer all questions as precisely as possible.

How important was the following for your decision to contact the out-of-hours service? *(Please tick only one box in each row)*

| **Own assessment and expectations** | **Importance** | | | | |  |
| --- | --- | --- | --- | --- | --- | --- |
| **Please rate the importance of the following:** | **No** | **Little** | **Some** | **Great** | **Huge** | **Not relevant** |
| … I thought that I needed quick advice or treatment by a physician |  |  |  |  |  |  |
| … my symptoms were so unpleasant that I could not wait until my own GP’s opening hours in the daytime |  |  |  |  |  |  |
| … I thought that my problem was life-threatening |  |  |  |  |  |  |
| … I was worried |  |  |  |  |  |  |
| … I expected that an examination would be necessary (e.g. blood test, throat swap, X-ray) |  |  |  |  |  |  |
| … I expected that I needed to be seen by a specialist or admitted to hospital |  |  |  |  |  |  |
| … I thought that I needed an ambulance to be sent |  |  |  |  |  |  |
| … I needed to renew a prescription |  |  |  |  |  |  |

How important was the following for your decision to contact the out-of-hours service? *(Please tick only one box in each row)*

| **Perceived barriers and benefits** | **Importance** | | | | |  |  |
| --- | --- | --- | --- | --- | --- | --- | --- |
| **Please rate the importance of the following:** | **No** | **Little** | **Some** | **Great** | **Huge** | **Not relevant** |  |
| … I could not get through on the telephone to my own GP in the daytime |  |  |  |  |  |  |  |
| … I could not get an appointment with my own GP fast enough |  |  |  |  |  |  |  |
| … I did not have possibility to contact my own GP in the daytime (e.g. because of work) |  |  |  |  |  |  |  |
| … I found it easiest to contact this out-of-hours service |  |  |  |  |  |  |  |
| … my need for contact arose outside my own GP’s opening hours | |  |  |  |  |  |  |
| … It was important for me to get help quickly so that I could go to work next day |  |  |  |  |  |  |  |
| … It was important for me that my child got help quickly because of daycare attendance on the following day |  |  |  |  |  |  |  |

How important was the following for your decision to contact the out-of-hours service? *(Please tick only one box in each row)*

| **Previous experience and knowledge** | **Importance** | | | | |  |
| --- | --- | --- | --- | --- | --- | --- |
| **Please rate the importance of the following:** | **No** | **Little** | **Some** | **Great** | **Huge** | **Not**  **relevant** |
| … I had previously experienced that similar symptoms required prompt treatment/examination |  |  |  |  |  |  |
| … I previously had positive experiences with this out-of-hours service |  |  |  |  |  |  |
| … I thought that this out-of-hours service was the right place to go with the problem in question |  |  |  |  |  |  |
| … I wanted a different professional assessment than my own GP’s (second opinion) |  |  |  |  |  |  |

How important was the following for your decision to contact the out-of-hours services? *(Please tick only one box in each row)*

| **Needs and wishes** | **Importance** | | | | |  |
| --- | --- | --- | --- | --- | --- | --- |
| **Please rate the importance of the following:** | **No** | **Little** | **Some** | **Great** | **Huge** | **Not relevant** |
| … I did not really know what to do |  |  |  |  |  |  |
| … I did not know where else to call |  |  |  |  |  |  |
| … I wanted to talk to a physician |  |  |  |  |  |  |
| … I wanted to talk to a nurse |  |  |  |  |  |  |
| … I could not take responsibility for the problem myself |  |  |  |  |  |  |
| … I had no one to talk to about the problem |  |  |  |  |  |  |
| … I was advised to call the out-of-hours services by a non-medical person (e.g. family member, friend, passer-by, kindergarten teacher) |  |  |  |  |  |  |

**THE MOST IMPORTANT REASON**

What was **the most important reason** for you to call the out-of-hours service and not your own GP?

________________________________

**PERCEPTION AND USE OF THE HEALTH CARE SYSTEM**

How satisfied are you generally with the following parts of the health care system? *(Please tick only one box in each row)*

|  | **Very satisfied** | **Satisfied** | **Neither satisfied nor dissatisfied** | **Dis-satisfied** | **Very dis-satisfied** | **Don’t know/not relevant** |
| --- | --- | --- | --- | --- | --- | --- |
| Own GP |  |  |  |  |  |  |
| Out-of-hours services |  |  |  |  |  |  |
| Emergency room/acute care |  |  |  |  |  |  |
| 112 |  |  |  |  |  |  |

**BACKGROUND INFORMATION**

1. Place of birth *(Please tick only one box in each row)*

|  | **Denmark** | **Other *(please state country)*** |
| --- | --- | --- |
| Where were you born (which country)? |  | ______ |
| Where was your mother born (which country)? |  | ______ |
| Where was your father born (which country)? |  | ______ |

What is your current marital status? *(Please tick only one box)*

Single (not previously married or cohabiting)

Single (divorced, separated, broken committed relationship)

Single (widow, widower)

Committed relationship

Cohabiting

Married

Other. *Please describe:* ________________________________

Do you have children? *(Please tick only one box)*

Yes 🡪 Number of children: ________________

No

What is your highest attained education? *(Please tick only one box)*

None

Primary and lower secondary education

Upper secondary education (high-school or similar)

One or several short courses (e.g. semi-skilled courses and training, labour market courses)

Skilled worker in industry or business (e.g. apprentice, trainee or basic vocational training)

Short higher education of less than three years (e.g. social and health care assistant, technician, business economist

Medium-length higher education of 3-4 years (e.g. primary school teacher, journalist, social worker, physiotherapist)

Long higher education of 5 years or more (e.g. engineer, physician, psychologist)

Other*. Please describe:* ________________________________

What is your current job position? *(Please tick only one box)*

Employed

Unemployed

Pensioner (old age, disability or early retirement)

Stay-at-home parent (with no other work)

On leave (maternity/paternity, educational, compassionate or parental)

Student

Other. *Please describe:* ________________________________

In general, would you say your health is: *(Please tick only one box)*

Excellent

Very good

Good

Fair

Poor

Don’t know/Unsure
