# Supplementary material for: Patient motives for contacting out-of-hours care in Denmark: a cross-sectional study
Source: BMC Emerg Med. 2020 Mar 17;20:20. doi: 10.1186/s12873-020-00312-3 (PMC7079359; doi:10.1186/s12873-020-00312-3)
Supplement: Supplementary file 2 — Additional file 2. Abbreviation list of the motives. [file 12873_2020_312_MOESM2_ESM.docx]

**Appendix 2.** Abbreviation list of the motives

| **Motives (full wording)** | | **Motives (abbreviations)** |
| --- | --- | --- |
| **Own assessments and expectations** | |  |
| … I thought that I needed quick advice or treatment by a physician | | Perceived need for prompt action |
| … my symptoms were so unpleasant that I could not wait until my own GP’s opening hours in the daytime | | Unpleasant symptoms |
| … I thought that my problem was life-threatening | | Perceived condition to be life-threatening |
| … I was worried | | Worried |
| … I expected that an examination would be necessary (e.g. blood test, throat swap, X-ray) | | Expected need for examination |
| … I expected that I needed to be seen by a specialist or admitted to hospital | | Expected need for specialist care/hospital admission |
| … I thought that I needed an ambulance to be sent | | Expected need for ambulance |
| … I needed to renew a prescription | | Renewal of prescription |
| **Perceived barriers and benefits** | |  |
| … I could not get through on the telephone to my own GP in the daytime | Own GP not accessible during daytime |  |
| … I could not get an appointment with my own GP fast enough | Own GP no time available soon enough |  |
| … I did not have possibility to contact my own GP in the daytime (e.g. because of work) | No possibility to contact own GP during daytime |  |
| … I found it easiest to contact this out-of-hours service | Easiest option |  |
| … my need for contact arose outside my own GP’s opening hours | | Need arose outside office hours |
| … It was important for me to get help quickly so that I could go to work next day | Need for quick help because of work |  |
| … It was important for me that my child got help quickly in consideration of day-care the following day | Need for quick help because of daycare attendance |  |
| **Previous experience and knowledge** |  |  |
| … I had previously experienced that similar symptoms required prompt treatment/examination | | Previous experience with similar symptoms |
| … I previously had positive experience with this out-of-hours service | | Previous positive experience with this health care service |
| … I thought that this out-of-hours service was the right place to go with the problem in question | | Perceived most suitable health care service |
| … I wanted a different professional assessment than my own GP’s (second opinion) | | Needed second opinion |
| **Needs and wishes** | |  |
| … I did not really know what to do | | Did not know what to do |
| … I did not know where else to call | | Did not know where else to call |
| … I wanted to talk to a physician | | Wanted to talk to a physician |
| … I wanted to talk to a nurse | | Wanted to talk to a nurse |
| … I could not take responsibility for the problem myself | | Could not take responsibility |
| … I had no one to talk to about the problem | | Nobody to talk to |
| … I was advised to call the out-of-hours services by a non-medical person (e.g. family member, friend, passer-by, kindergarten teacher) | | Recommended (from non-medical person) to call |
